# Less is More: Nasogastric Tube Perforation in a Patient With Prior Roux‐en‐Y Gastric Bypass

**DOI:** 10.1155/crgm/2949430

**Published:** 2025-12-17

**Authors:** Mario Tavakoli, Sam Papasotiriou, Dustin R. Fraidenburg

**Affiliations:** ^1^ Department of Internal Medicine, University of Illinois-Chicago, Chicago, Illinois, USA, uic.edu

**Keywords:** gastric perforation, nasogastric tube, procedural complications, Roux-en-Y gastric bypass

## Abstract

Nasogastric (NG) tube placement is a routine inpatient procedure that is generally considered safe. NG perforation is a rare complication, but when it occurs, it is often serious or even deadly. The risk for perforation is increased in patients with a history of connective tissue disorders, inflammatory bowel disease, or prior abdominal surgeries such as Roux‐en‐Y gastric bypass (RYB). We present a fatal case of a 51‐year‐old woman with a recent RYB who suffered an NG‐tube perforation leading to peritonitis and septic shock. This case highlights the extreme care healthcare providers must have when placing NG tubes in patients with prior abdominal surgeries, while offering suggestions on how to minimize the risk of fatal complications.

## 1. Introduction

Nasogastric (NG) tube perforation is a serious but rare complication that can occur in the hospital setting. Although specific incidence rates for gastric perforations are not well documented, population studies have estimated that the annual incidence rate of esophageal perforation from NG tubes is 3.1 per 1,000,000 cases [1]. While gastric perforations resulting from NG tube placement are exceedingly rare, they are more common in neonates and infants than in adults. The risk for perforation is increased in patients with a history of connective tissue disorders, inflammatory bowel disease, or prior abdominal surgeries such as Roux‐en‐Y gastric bypass (RYB). There are approximately 10 reported cases in the literature of gastric perforations from NG tubes in adults [2]. Our case describes a 51‐year‐old woman with a history of a recent RYB procedure who experienced gastric perforation following NG tube placement in the intensive care unit (ICU). Although the providers were able to promptly identify the cause of her acute decompensation, the patient eventually died secondary to peritonitis and septic shock. This case highlights the risk of placing NG tubes in patients with prior bariatric procedures, while offering suggestions on how to prevent fatal complications in this patient population.

## 2. Case Presentation

A 51‐year‐old woman presented as a transfer from an outside hospital for further work‐up of a non–ST‐elevation myocardial infarction (NSTEMI) and worsening heart failure. Her past medical history included heart failure with a reduced ejection fraction (EF), cocaine use disorder, opioid use disorder on methadone, generalized anxiety disorder, and prior sleeve gastrectomy with recent conversion to a RYB 71 days prior to admission. The patient was found to have a severely reduced EF of 15%–20%, with severe hypokinesis on a transthoracic echocardiogram (TTE) and an elevated troponin, although no ischemic changes were noted on an electrocardiogram (EKG). She was given high‐dose aspirin and atorvastatin and was initiated on a heparin drip. A left heart catherization showed no evidence of coronary artery disease; therefore, the heparin drip was discontinued. A repeat TTE revealed an improved EF of 40%–45%, which was unchanged from her previous TTE 5 months prior.

**Figure 1:**
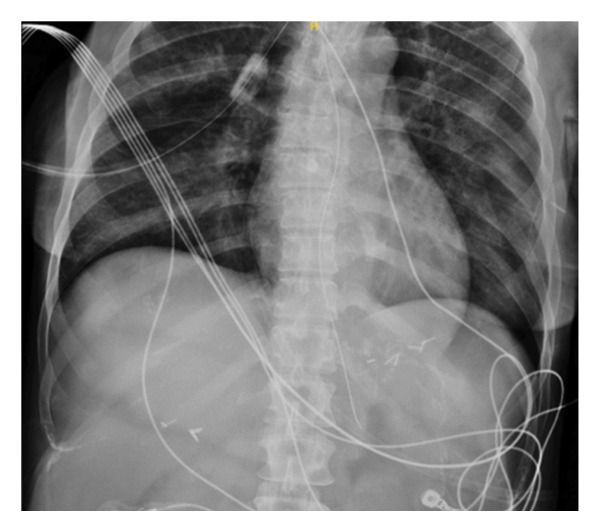
Postplacement abdominal X‐ray confirming that the NG tube was in a satisfactory position.

The patient initially had a NG tube placed on hospital Day 1, and an abdominal X‐ray ordered for confirmation of placement was read as “NG tube with tip in the distal esophagus at the gastroesophageal (GE) junction, should be advanced by 6 to 7 cm into the stomach.” After repositioning, the abdominal X‐ray was read as “NG tube is in place with side hole still superior to the diaphragm and the tip at the GE junction, recommend 5 to 6 cm of advancement.” The ICU team then obtained a chest X‐ray to assess for NG tube coiling, and imaging results did not show any evidence of coiling. At this time, the length of insertion of the NG tube was documented at 86 cm. The NG tube was replaced, and the abdominal X‐ray was read as “the side hole of the NG tube in the GE junction, recommend to advance by at least 2 cm.” On hospital Day 3, an abdominal X‐ray confirmed that the NG tube was in place, and the length of insertion was noted to be at 53 cm (Figure 1).

**Figure 2:**
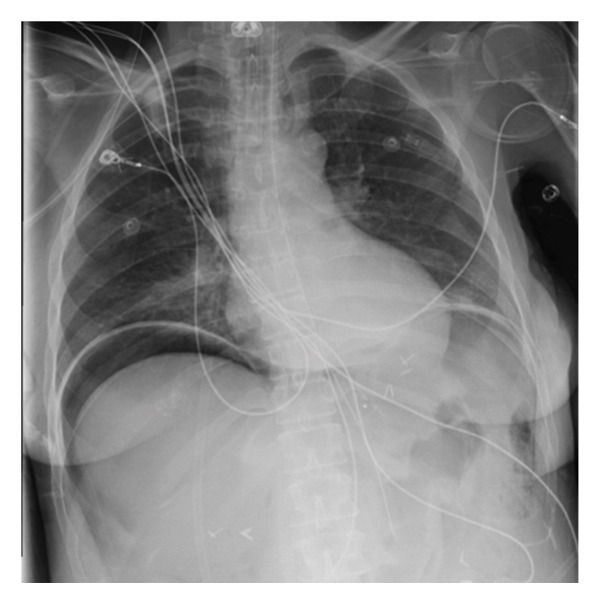
Urgent chest X‐ray showing bilateral, large‐volume pneumoperitoneum.

During the morning of hospital Day 4, the patient began developing abdominal distension with increasing pressor and oxygen requirements, prompting the ICU team to obtain an urgent chest X‐ray which revealed a large‐volume pneumoperitoneum (Figure 2). Concerned for a suspected perforated viscus, the patient was taken emergently to the operating room with general surgery. During a diagnostic laparoscopy, they found a gastric pouch perforation which they subsequently repaired. The surgery team visualized the NG tube penetrating a 1‐cm perforation, with tube feeds throughout the abdomen.

The patient was then transferred back to the ICU, where she continued to decompensate with multiorgan failure in the setting of septic shock. Stress dose steroids were initiated, and her antibiotic regimen was further broadened. Continuous venovenous hemofiltration (CVVH) and a bicarbonate drip were initiated for persistent lactic acidosis. A bedside TTE revealed an acutely reduced EF; therefore, cardiology was consulted for a possible component of cardiogenic shock.

Shortly after, the patient experienced ventricular tachycardia and expired on hospital Day 6.

## 3. Discussion

Although an exceedingly rare complication, NG tube perforation is associated with significant morbidity and mortality. Patients with prior abdominal surgeries, such as RYB, are at an increased risk of perforation due to the reduced area for enteral feeding caused by the surgical anatomy of the bypass. Studies have shown that the use of the corrected nose–earlobe–xiphoid (CoNEX) method is exceptionally accurate in predicting the length of NG tube insertion in adults [3]. The formula includes the distance from the tip of the nose to the earlobe to the xiphoid process in centimeters, in addition to a correction factor of 30.76 cm plus 6 cm to ensure that the tip is placed within the stomach [3]. This calculation averages 58 cm in patients with standard anatomy, with the consideration that 6–8 cm should be within the stomach to avoid aspiration of gastric contents. Patients with a prior history of a RYB, however, have gastric pouches that on average measure 4 to 5 cm [4]. There are also high‐risk anatomical structures in the area that increase the risk of perforation, such as the gastrojejunal anastomosis [4]. This narrow anastomosis between the gastric pouch and jejunum is often poorly vascularized, increasing the risk of injury and ulceration [5]. While it is not well documented in the literature, the insertion of NG tubes is not an absolute contraindication in patients with a history of RYB. However, a review of discussion boards on bariatric surgery revealed that NG tube placement is generally discouraged in the immediate postoperative period due to the increased risk of perforation and staple line disruption [6]. Although this risk decreases after several weeks once the staple lines heal, NG tubes should be placed with extreme caution [6]. While the patient in our case had her RYB revision approximately 2 months prior to her presentation, it seems that some patients are advised by their bariatric surgeon to decline having NG tubes placed indefinitely owing to the increased risk of complications [7].

In patients with normal anatomy, there are no perceived sites in the stomach that are highly vulnerable to perforation, as the organ is highly distensible and can withstand an air volume greater than 1.6 L [7]. The volume of a gastric pouch following a RYB, however, is approximately 30 mL [7]. The average length of an adult NG tube is approximately 125 cm, with the distal 6–8 cm containing side holes that allow aspiration or infusion of fluids. While patients with typical gastric anatomy are able to tolerate coiling of the NG tube with little risk of causing significant damage, patients with a history of RYB do not have room for the tube to coil. Furthermore, given that the average height of a gastric pouch following RYB is approximately 4 cm, the distal side holes are unlikely to be contained within the pouch simultaneously. In most cases, either the proximal side holes of the NG tube would be in the lower esophagus, or the distal holes would enter the Roux limb past the gastrojejunal anastomosis. Nutrient solutions infused into the esophagus increase the risk of aspiration, whereas tubes traversing the anastomosis increase the risk of perforation. Serosal adhesions that often develop after abdominal surgeries can also cause kinks in the Roux limb, further increasing the risk of perforation if the tube is advanced past the anastomosis [7].

With all of this in mind, we have several suggestions to prevent NG tube perforation in patients with a history of gastric bypass. First and foremost, it is paramount that all team members understand the altered anatomy in this subset of patients. Nurses and physicians who are tasked with inserting NG tubes in these patients should be extremely cautious and avoid advancing the tube against resistance. Moreover, it appears that the radiologists who read the abdominal X‐rays in our case were not alerted of the patient’s history of a prior RYB. It is essential that radiologists who interpret NG tube positions in these patients are made aware of prior abdominal surgeries that may influence the length of insertion. According to the literature, it is believed that the length of NG tubes inserted in patients with a prior RYB should be 10 to 15 cm shorter than that in patients with normal gastric anatomy [7]. Other options that may be considered include endoscopic placement of nasojejunal tubes or percutaneous endoscopic jejunostomies, if the patient is likely to require enteral feedings for a longer period of time. Placement under the jurisdiction of interventional radiology (IR) has also been shown to reduce the risk of perforation and ensure satisfactory placement of feeding tubes [8]. These findings all strengthen the argument that there should be a lower threshold for endoscopic or IR‐guided placement of enteral feeding tubes in these patients. This case highlights the importance of multidisciplinary communication when NG tube placement is considered in patients with a history of bariatric surgery. Although these instances are generally uncommon, hospitals should develop protocols to address this issue in an attempt to avoid the significant risk and fatal complications that may arise.

**Nomenclature**
CVVH: Continuous venovenous hemofiltration
CoNEX: Corrected nose–earlobe–xiphoid
EF: Ejection fraction
EKG: Electrocardiogram
GE: Gastroesophageal
ICU: Intensive care unit
IR: Interventional radiology
NG: Nasogastric
NSTEMI: Non–ST‐elevation myocardial infarction
RYB: Roux‐en‐Y bypass
TTE: Transthoracic echocardiogram

## Ethics Statement

This retrospective case report was exempted from the University of Illinois Chicago Institutional Review Board oversight.

## Consent

No written consent has been obtained from the patient as there are no patient identifiable data included in this case report.

## Disclosure

All of the authors have read the manuscript and approved its submission.

## Conflicts of Interest

The authors declare no conflicts of interest.

## Author Contributions

Mario Tavakoli completed the introduction, discussion, and final revisions. Sam Papasotiriou described the case presentation. Dustin R. Fraidenburg contributed to the editing of the manuscript.

## Funding

This manuscript was funded by the University of Illinois—Chicago.

## Data Availability

Data sharing is not applicable to this article as no new data were created or analyzed in this study.
